# Studies on New Activities of Enantiomers of 2-(2-Hydroxypropanamido) Benzoic Acid: Antiplatelet Aggregation and Antithrombosis

**DOI:** 10.1371/journal.pone.0170334

**Published:** 2017-01-20

**Authors:** Qili Zhang, Danlin Wang, Meiyan Zhang, Yunli Zhao, Zhiguo Yu

**Affiliations:** School of Pharmacy, Shenyang Pharmaceutical University, Shenyang, China; Royal College of Surgeons in Ireland, IRELAND

## Abstract

*R*-/*S*-2-(2-Hydroxypropanamido) benzoic acid (*R-/S-*HPABA), a marine-derived anti-inflammatory drug, however, the antiplatelet and antithrombotic effects have not been investigated. In this paper, the *in vitro* antiplatelet activities and *in vivo* antithrombotic effects of *R-*/*S-*HPABA were investigated, for the first time. The effects of *R-*/*S-*HPABA on platelet aggregation induced by adenosine diphosphate (ADP), collagen (COLL) and arachidonic acid (AA) were evaluated. In addition, the *in vivo* bleeding time, clotting time, collagen-epinephrine induced pulmonary thrombosis and common carotid artery thrombosis were also investigated in rats. *R-*/*S-*HPABA significantly inhibited ADP, COLL and AA induced platelet aggregation in rabbit platelet rich plasma *in vitro* compared with control group, to a degree similar to that of aspirin. Besides, *R-*/*S-*HPABA prolonged bleeding time and clotting time as well as increased the recovery rate obviously in pulmonary thrombosis. Moreover, the level of thromboxane B_2_ (TXB_2_) was decreased while the production of 6-keto-prostaglandin F1α (6-keto-PGF1α) was increased markedly by *R-*/*S-*HPABA. Furthermore, *R-*/*S-*HPABA reduced carotid artery thrombosis weight. These results illustrated that *R-*/*S-*HPABA could be a potent antiplatelet aggregation and antithrombotic agent.

## Introduction

Thrombosis is that the blood form clots accompanied by insoluble fibrin and deposition of platelets on spalling or repaired surface of the cardiovascular system [1–3]. Efficient clotting limits the loss of blood at an injury site, whereas inappropriate formation of thrombin in veins or arteries is a common cause of disability and death [4, 5]. Abnormal intravascular thrombosis may result in a variety of cardiovascular diseases such as stroke, pulmonary embolism, myocardial infarction, unstable angina, atherosclerotic plaque rupture, atrial fibrillation, restenosis, renal damage and deep vein thrombosis [5, 6–10]. To the best of our knowledge, platelets play a major part not only in normal hemostasis but also in the pathogenesis of thrombosis. Accordingly, platelet activation make significant effects on initiation of haemostasis, thrombosis and various cardiovascular and cerebrovascular diseases [11, 12]. It is imperative to control platelets function in preventing and treating thrombosis events [13]. In addition, most thromboembolic processes require anticoagulant therapy. The situation further emphasizes the importance of developing potent anticoagulant and anti-thrombosis agents [14].

Various anti-thrombotic drugs, such as antiplatelets (aspirin, clopidogrel and tirofiban), anticoagulant drugs (heparin, low molecular weight heparins and argatroban) and fibrinolytic drugs (urokinase, pro-urokinase and reteplase) have been developed and used clinically for their effects to prevent the development of thrombosis or its recurrence. Among them, aspirin and clopidogrel, are well recognized clinically to prevent platelet-associated diseases including thrombosis and atherosclerosis [15, 16]. Although intensive research in the thrombosis field, narrow therapeutic window, bleeding risk, incidence of resistance, and unwanted drug interactions are the cause of major concern [17–19]. Thus there is still a need for the development of antithrombotic drugs with minimum side effects and better efficacy.

**Fig 1:**
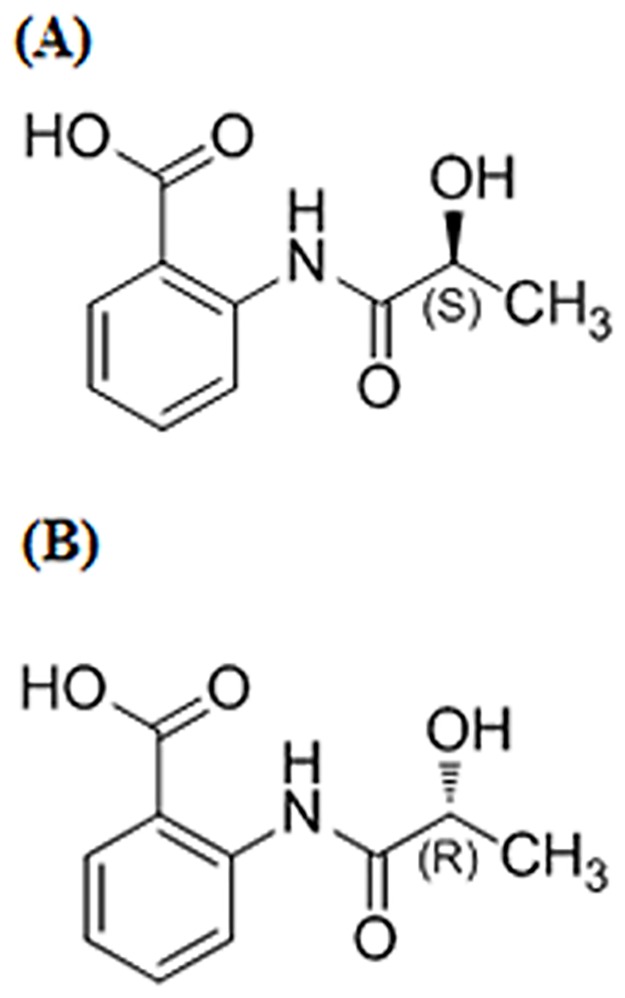
Chemical structures of *S*-HPABA (A) and *R*-HPABA (B).

*R*-/*S*-2-(2-Hydroxypropanamido) benzoic acid (*R-/S-*HPABA) (Fig 1), with a single stereogenic carbon atom and exists in two enantiomeric forms, which was initially isolated from the fermentation broth of a marine fungus *Penicillium chrysogenum* by our group [20]. *R-/S-*HPABA possessed the similar structure with aspirin. Moreover, it was found that *R-/S-*HPABA presented remarkable analgesic and anti-inflammatory activities, but did not have ulcerogenic effects like aspirin [20, 21]. Therefore, we carried out further researches on antiplatelet aggregation and antithrombus activities of *R-/S-*HPABA.

In this paper, we examined the crucial role of *R-/S-*HPABA on platelet aggregation *in vitro* and on thrombosis formation *in vivo*. Furthermore, we assessed their effects on the bleeding time, clotting time, and collagen-epinephrine induced pulmonary thrombosis. In addition, the possible antiplatelet aggregation mechanism of *R-/S-*HPABA was also investigated firstly. These results are beneficial to the further study of the function of *R-/S-*HPABA on cardiovascular and cerebrovascular diseases.

## Materials and Methods

### Materials and reagents

*R*-HPABA (optical purity > 99.3%) and *S*-HPABA (optical purity > 98.8%) were synthesized in School of Pharmacy, Shenyang Pharmaceutical University (Shenyang, China). Aspirin (purity ≥ 99.5%) was supplied by Shandong Xinhua Pharmaceutical Co., Ltd (Shandong, China). Adenosine diphosphate (ADP), collagen (COLL, Type I) and arachidonic acid (AA) were purchased from Shanghai Ryon Biological Technology Co., Ltd (Shanghai, China). Epinephrine hydrochloride injection was obtained from Grandpharma (China) Co., Ltd. Trisodium citrate and dimethyl sulfoxide (DMSO) were of analytical grade. Thromboxane B_2_ (TXB_2_) ELISA kit, 6-keto-prostaglandin F1α (6-keto-PGF1α) ELISA kit, cyclooxygenase-1(COX-1) ELISA kit and lactate dehydrogenase (LDH) kit were purchased from Nanjing Jiancheng Bioengineering Institute Co., Ltd. (China). Deionized water was purified using a Milli-Q system (Millipore, Milford, MA, USA).

### Animals

New Zealand white male rabbits (2.0–2.5 kg, 3 months of age) (No. 211001600000401), Male Kunming mice (weight approximately 18–22 g, 5–6 weeks of age) (No. 211002300011325) and Sprague-Dawley rats (male, 220–250 g, 6–8 weeks of age) (No. 211002300011732) were purchased from Experimental Animal Center of Shenyang Pharmaceutical University (Shenyang, China). The animals were housed in standard environmental conditions with standardized temperature (22 ± 2°C), humidity (50 ± 10%) and a natural light/dark cycle and fed a standard diet with water adlibitum. The rats were fasted for 12 h, but allowed free access to water before the experiment was carried out.

### Ethics statement

All experimental procedures were carried out according to the Guideline for Animal Experimentation of Institutional Animal Care and Use Committee of Shenyang Pharmaceutical University, and the protocol was reviewed and approved by the Animal Ethics Committee of the Shenyang Pharmaceutical University. All surgery was performed under urethane anesthesia, and all efforts were made to minimize suffering.

### Preparation of test drugs and agonists

Solutions of *R-/S*-HPABA and aspirin were prepared in DMSO at 0.15 mg/mL. ADP, AA and COLL were prepared in DMSO at 5 mmol/L, 20 mmol/L and 1 mg/mL, respectively.

### Preparation of platelet-rich plasma (PRP) and platelet-poor plasma (PPP)

Blood was collected from the ear margin vein of rabbits and dispensed into centrifuge tubes containing trisodium citrate (3.8%, 1: 9, *v/v*). Then, blood samples were centrifuged at 1000 r/min for 10 min at room temperature and the supernatant PRP was isolated. The residue was centrifuged at 3000 r/min for 10 min at room temperature to obtain the PPP.

### *In vitro* platelet aggregation assay

The platelet aggregation rates were determined following the Born’s turbidimetric method [22] on an LG-PABER-I platelet aggregator (Beijing Steellex Tech Instru Co., Ltd. China). The percent change in light transmission was recorded to indicate the aggregation rate: PRP and PPP were used to set the the baseline and maximal transmission, respectively. The platelet number was determined by an automatic counter (6.7×10^8^ cell/mL) and PRP was adjusted to 3 ×10^8^/mL with PPP. PRP samples (200 μL) with *R-*/*S-*HPABA at a concentration of 0.15 mg/ml (5μL) were added into the microbasin. PRP samples were pre-incubated at 37°C for 2 min in the aggregometer, and then 20 μL of ADP (AA, COLL) was added to the PRP samples to induce acute thrombosis. In blank and positive control experiments, DMSO (5μL) and aspirin (5μL) were added instead of the test samples, respectively. Tests were performed within 3 h to avoid platelet inactivation. The percent inhibition was used to evaluate the effects of *R-*/*S-*HPABA and aspirin compared with blank control samples. Maximum aggregation was recorded for the blank control (CA) and the different tests (TA). The inhibition rate (IA) was calculated as: IA (%) = (CA-TA) / CA ×100% [23].

### Determination of cytotoxicity in rat PRP

Lactate dehydrogenase (LDH) is a well-established indicator of cell viability. Therefore, cytotoxic effects of *R-*/*S-*HPABA on platelets were evaluated by measuring lactate LDH leakage [24, 25]. After various times of incubation with 150 μg/mL *R*-/*S*-HPABA, PRP was centrifuged. In the present studies, the measurement of LDH release was performed using a LDH cytotoxicity kit according to manufacturer’s instructions with minor modifications. The extent of LDH leakage was expressed as percentage of total enzyme activity measured in a control incubation lysed with 0.3% Triton X-100.

### Experiments in mice

Male Kunming mice were divided into eight groups of eight or ten rats in each group as follows: aspirin 100 mg/kg (positive control group), *R-*/*S-*HPABA low 50 mg/kg, medium 100 mg/kg, high 200 mg/kg dose and sodium carboxymethyl cellulose (blank control group). All drugs were given orally once daily for 7 days, dissolved in sodium carboxymethyl cellulose (CMC-Na). After the last administration, mice were anesthetized with urethane (1 g/kg, i.p.).

#### Bleeding time

Mouse tail-bleeding time was measured as described previously [26]. One hour after the last administration, mice were anesthetized with urethane and placed on a 37°C heating pad. The tail was transected 5 mm from the tip and immediately immersed in saline at 37°C. Timing was started when the blood flowed and bleeding time (including those of re-bleeding) was recorded within a 15 min period.

#### Clotting time

One hour after last treatment administration, mice were anesthetized with urethane. Blood was collected from fosse orbital vein with glass capillary and two drops of blood were dropped at the end of a slide. Timing immediately started with a stopwatch. One of the drops of blood was pricked lightly with a pin every 30 s. Timer was stopped when a blood streak appeared. The other drop of blood was used for retesting [27].

#### Collagen-epinephrine induced pulmonary thrombosis

One hour after the last drug administration, a mixture of collagen (500 μg/kg) plus epinephrine (50 μg/kg) was injected to the tail vein of mice to induce acute thrombosis [28, 29]. Each mouse was carefully examined to determine whether the mouse was paralyzed, dead, or recovered from the acute thrombotic challenge within 15 min. The inhibitory rate was computed using the following formula: Inhibition (%) = ((number of deaths in controls—number of deaths in treated)/number of deaths in controls) ×100%.

### Experiments in rats

The male Sprague-Dawley rats were divided into groups of five rats in each group and treated as that of mice.

#### Common carotid artery thrombosis

Carotid artery thrombosis was measured as described with minor modifications [30, 31]. One hour after the last administration, rats were anesthetized with 20% urethane (0.5 mL/100 g i.p.) and placed on heated surface and under a heating lamp to be maintained at 37°C. An incision was made of about 3 cm in the midline on the throat and the left carotid artery was bluntly isolated carefully. The artery near to the heart was clamped with artery clamp. Take a 12 fine needle attached a thread, with a knot at the end of the thread, through the common carotid artery from the part near to heart to the part far from heart. Then slowly loosen the artery clamp, restore blood flow. The incisions were kept moist throughout the experiment by gently applying buffer using cotton wool balls saturated with warm saline to avoid disturbance of the circulatory system. Three hours after surgery and an injured carotid artery segment (1.0 cm) was then cut off and placed on the filter paper to dry and was then weighed. The rate of thrombosis inhibition was calculated as: Inhibition rate (%) = (A- A_1_)/A×100%, where A was the wet weight of the thrombus in the control group and A_1_ was that in agents treated groups.

#### Measurement of the plasma concentration of TXB_2_ and 6-keto-PGF1α

Forty male Sprague-Dawley rats were divided into groups and treated as above in the arterial thrombosis model. Three hours after surgery, the abdominal aorta was isolated and the blood was collected by abdominal aortic puncture using 5 mL syringe with trisodium citrate (3.8%, 1: 9, *v/v*). The PRP samples were prepared as above in section “Preparation of platelet-rich plasma (PRP) and platelet-poor plasma (PPP)”. Preparation of platelet-rich plasma (PRP) and platelet-poor plasma (PPP). The plasma was separated and stored at -20°C before the determination. The plasma concentrations of TXB_2_ and 6-keto-PGF1α were measured by enzyme-linked immunoassay (ELISA).

#### Measurement of cyclooxygenase-1(COX-1) activity

Twenty male Sprague-Dawley rats were divided into four groups of five rats in each group as follows: aspirin 100 mg/kg (positive control group), *R-*HPABA (200 mg/kg), *S*-HPABA (200 mg/kg) and sodium carboxymethyl cellulose (CMC-Na) (blank control group). All drugs were given orally once daily for 7 days. One hour after the last administration, the abdominal aorta was isolated and the blood was collected. The PRP samples were prepared as above mentioned and stored at -20°C before the determination. The measurement of COX-1 activity was performed using a COX-1 activity assay kit according to the manufacturer’s instruction.

### Histopathology

At the end of the common carotid artery thrombosis experiment, the carotid artery segments from both sides were excised and soaked in 10% neutral buffered formaldehyde solution. After conventional dehydration, paraffin embedding, slice, and haematoxylin and eosin (HE) staining, the pathological changes of carotid artery segments were observed.

### Statistical analysis

All the results are presented as mean values ± standard deviation (mean ± SD). Differences between control and treatment groups were determined using one way ANOVA for comparison between three or more groups. Student’s t-test was used to determine statistical significance when two groups of data were compared. All of the statistical analyses were performed using SPSS Statistics 17.0. *p* < 0.05 and *p* < 0.01 were considered significant and dramatic significant, respectively.

## Results

### *In vitro* platelet aggregation assay

**Fig 2:**
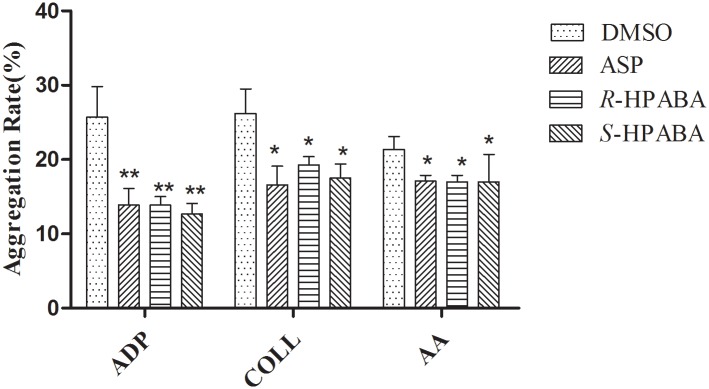
The effects of *R-*/*S-*HPABA and aspirin on the platelet aggregation induced by ADP, Coll and AA. *p < 0.05 vs. DMSO group, **p < 0.01 vs. DMSO group.

*R-*/*S-*HPABA could significantly inhibit the platelet aggregation induced by ADP (5 mmol/L), AA (20 mmol/L) and COLL (1 mg/mL) compared with the control group (*p* < 0.05). These results suggest that *R*-/*S*-HPABA has an effect on platelet aggregation, especially on ADP-induced platelet aggregation. In addition, *R-*/*S-*HPABA had the same effect on platelet aggregation compared with aspirin at the same concentration of 0.15 mg/mL (*p* > 0.05). The results are shown in Fig 2.

### Determination of cytotoxicity in rat PRP

**Fig 3:**
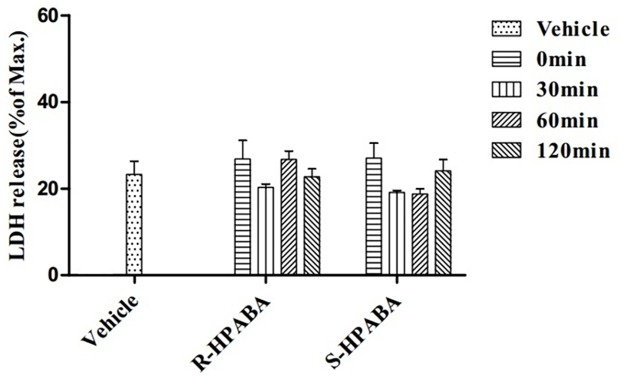
Effects of *R-*/*S-*HPABA on LDH release from platelets.

When platelets were exposed to *R-*/*S-*HPABA (150 μg/mL), there were no significant changes to the release of lactate dehydrogenase (LDH) compared with vehicle (*p* > 0.05). The results are presented in Fig 3, suggesting that the inhibitory effect of *R-*/*S-*HPABA on platelet function was not due to cytotoxicity.

### Experiments in mice

#### Bleeding time and clotting time

All the doses of *R-*/*S-*HPABA (50, 100 and 200 mg/kg) significantly prolonged the bleeding time (*p* < 0.01) and clotting time (*p* < 0.05) compared with the control group. There were no significant differences between aspirin and *R-*/*S-*HPABA group. Besides, *R*-HPABA and *S*-HPABA had about the same effect on bleeding time and clotting time. No dose-dependently was, moreover, observed for *R-*/*S-*HPABA. The results are shown in Table 1.

**Table 1:** Effect of *R-*/*S*-HPABA, aspirin on bleeding time and clotting time, (mean ± SD) after 7 days of oral treatment in mice (n = 8).

Group	Dose (mg/kg)	Bleeding time (min)	Clotting time (s)
CMC-Na	-	3.57 ± 0.46	221 ± 42.6
Aspirin	100	10.12 ± 0.35*	244 ± 24.7*
*R-*HPABA (low dose)	50	8.35 ± 1.20*	229 ± 19.6*
*R*-HPABA (medium dose)	100	11.04 ± 0.76*	247 ± 26.1*
*R*-HPABA (high dose)	200	8.26 ± 0.44*	235 ± 25.4*
*S*-HPABA (low dose)	50	8.28 ± 1.20*	227 ± 23.4*
*S*-HPABA (medium dose)	100	9.50 ± 0.60*	230 ± 24.4*
*S*-HPABA (high dose)	200	8.15 ± 0.85*	227 ± 15.4*

*p < 0.05 vs. control group

#### Collagen-epinephrine induced pulmonary thrombosis

As presented in Table 2, *R-*/*S-*HPABA significantly increased the recovery rate compared with the control group (*p* < 0.01). Compared with the aspirin group, the medium and high dose of *R*-HPABA (100, 200 mg/kg) and the high dose of *S*-HPABA (200 mg/kg) significantly increased the inhibitory rate (*p* < 0.05). In addition, the effects of *R*-HPABA at medium and high dose were significantly higher than that of *S*-HPABA at the same concentration.

**Table 2:** Effects of *R*-/*S*-HPABA, aspirin on collagen—epinephrine induced pulmonary thrombosis (mean ± SD) after 7 days of oral treatment in mice (n = 10).

Group	Dose(mg/kg)	No. Recovery	Recovery rate (%)
CMC-Na	-	0	0
Aspirin	100	4	40.0
*R-*HPABA (low dose)	50	2	20.0
*R*-HPABA (medium dose)	100	6	60.0*^#^
*R*-HPABA (high dose)	200	7	70.0*^#^
*S*-HPABA (low dose)	50	1	10.0
*S*-HPABA (medium dose)	100	3	30.0
*S*-HPABA (high dose)	200	5	50.0*

*p < 0.05 vs. aspirin group.

^#^p < 0.05 vs. *S*-HPABA group at same concentrations.

### Experiments in rats

#### Common carotid artery thrombosis

*R-*/*S-*HPABA reduced the weight of thrombus significantly compared with the control group (*p* < 0.01). The effects of *R-*/*S-*HPABA on thrombus weight were not very different from that of aspirin treatment (*p* > 0.05). Therefore, the effects of *R*-HPABA and *S*-HPABA were similar on thrombus weight. Both parameters are presented in Table 3.

**Table 3:** Effect of *R*-/*S*-HPABA, aspirin on common carotid artery thrombosis in rats (mean ± SD, n = 5).

Group	Dose (mg/kg)	Weight of thrombus (mg)	Inhibitory rate (%)
CMC-Na	-	3.60 ± 0.14	-
Aspirin	100	2.05 ± 0.17*	43.1
*R-*HPABA(low dose)	50	2.16 ± 0.23*	40.0
*R*-HPABA(medium dose)	100	1.90 ± 0.10*	47.2
*R*-HPABA(high dose)	200	2.10 ±0.51*	41.7
*S*-HPABA(low dose)	50	2.10 ± 0.30*	41.7
*S*-HPABA(medium dose)	100	2.14 ± 0.18*	40.6
*S*-HPABA(high dose)	200	2.16 ± 0.23*	40.0

*p < 0.01 vs. control group

#### Measurement of the plasma concentration of TXB_2_ and 6-keto-PGF1α

As illustrated in Table 4, compared with the control group, the arterial plasma concentration of 6-keto-PGF1α was significantly increased (*p* < 0.05) and that of TXB_2_ was decreased significantly (*p* < 0.05) in the *R-*/*S-*HPABA group but with no dose-dependence. In addition, the effects of *R*-HPABA were not significantly different from that of *S*-HPABA. *R*-HPABA (low and medium dose) and *S*-HPABA (medium dose) had about the same effects as the aspirin (100 mg/kg) for increase of 6-keto-PGF1α and decrease of TXB_2_.

**Table 4:** Effect of *R*-/*S*-HPABA, aspirin on the plasma concentrations of TXB_2_ and 6-keto-PGF1α in rats (mean ± SD, n = 5).

Group	Dose (mg/kg)	TXB_2_ (pg/mL)	6-keto-PGF1α (pg/mL)
CMC-Na	-	156.0 ± 18.6	93.0 ± 30.1
Aspirin	100	60.0 ± 26.6*	73.8 ± 28.2
*R-*HPABA(low dose)	50	88.0 ± 40.7*	168.0 ± 55.5*
*R*-HPABA(medium dose)	100	61.0 ± 7.7*	154.0 ± 20.6*
*R*-HPABA(high dose)	200	62.0 ±15.4*	154.0 ± 37.9*
*S*-HPABA(low dose)	50	78.0 ± 10.7*	165.0 ± 22.3*
*S*-HPABA(medium dose)	100	68.0 ±14.4*	148.0 ± 31.1*
*S*-HPABA(high dose)	200	94.0 ± 9.7*	154.0 ± 22.3*

*p < 0.05 vs. control group.

#### Measurement of cyclooxygenase-1(COX-1) activity

**Fig 4:**
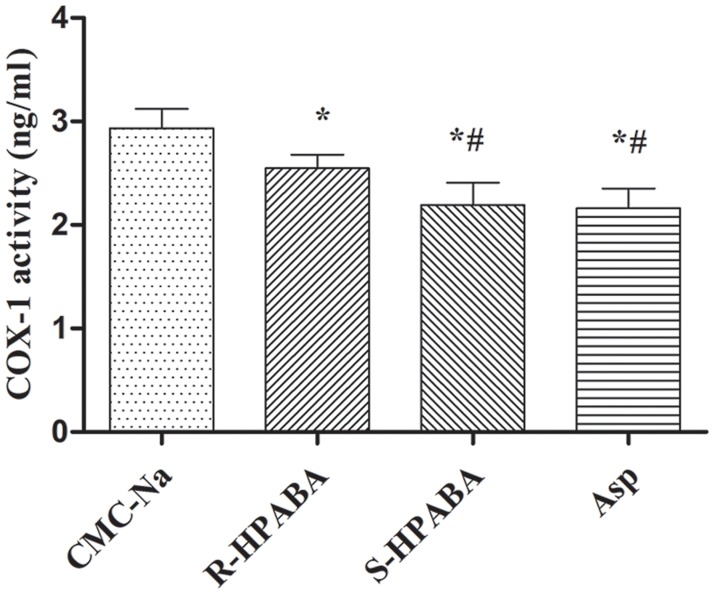
Effects of *R-*/*S-*HPABA on cyclooxygenase-1(COX-1) activity. *p < 0.01 vs. CMC-Na group, ^#^p < 0.01 vs. *R*-HPABA group.

As presented in Fig 4, compared with the control group, *R-*/*S-*HPABA and aspirin significantly inhibit the activity of COX-1 (*p* < 0.01). *S*-HPABA had about the same effects as the aspirin for the inhibition of COX-1 activity. Additionally, the effect of *R*-HPABA on the inhibition of COX-1 activity was not higher than that of *S*-HPABA.

### Histopathology

**Fig 5:**
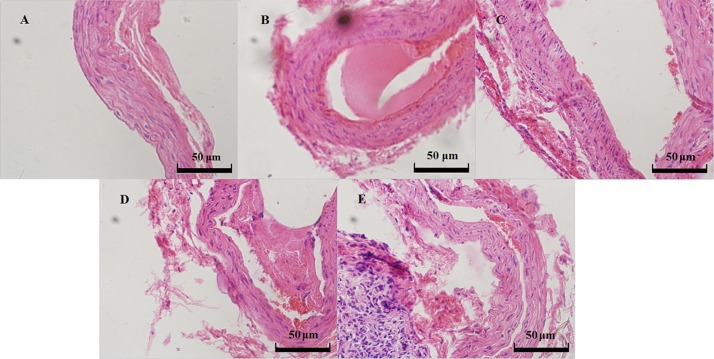
Light micrographs of common carotid artery thrombosis from the control group (B), 100 mg/kg aspirin group (C), 100 mg/kg *R*-HPABA group (D) and 100 mg/kg *S*-HPABA group (E). A: the normal common carotid artery.

*R-*/*S-*HPABA and aspirin significantly inhibit the formation of thrombus compared with control group. Transparent thrombosis could be observed obviously in the carotid artery in control group. The results are presented in Fig 5.

## Discussion

The injured vessels can result in the adhesion, activation and aggregation of platelets. Endogenous agonists, such as ADP, collagen, thrombin and TXA_2_ [32, 33] are then amplify this effect. ADP is one of the most import mediators of aggregation. The combination of ADP and purinergic receptors (P2Y_1_ and P2Y_12_) alter the platelet shape and promote aggregation [34]. In addition, Ca^2+^ and fibrinogen are involved in ADP-induced aggregation. The intracellular calcium mobilization and platelet shape conversion are activated by the P2Y_1_ receptor pathway. Whereas platelet aggregation is extended by the P2Y_12_ receptor pathway. In present studies, the results showed that *R*-/*S*-HPABA could significantly inhibit the platelet aggregation, especially ADP-induced platelet aggregation, moreover, this effect were comparable with that of aspirin. In addition, the inhibitory effect of *R-*/*S-*HPABA on platelet aggregation was not due to cytotoxicity.

The quantity and function of the platelets and the function of capillary are the major factors in the duration of bleeding time [35]. As shown in this study, compared with the control group, all the doses of *R-*/*S-*HPABA slightly prolonged the bleeding time in mice tails (*p* < 0.05), suggesting that *R-*/*S-*HPABA might inhibit platelet function and capillary systole. Meanwhile, significant antiplatelet aggregation *in vitro* and antithrombotic effects *in vivo* for *R-*/*S-*HPABA were seen in present study. Therefore, *R-*/*S-*HPABA are relatively safe for inhibiting platelet aggregation at the effective concentrations.

Clotting time is the time from when the blood is isolated from the body to coagulation, which is related to the extrinsic pathway. The duration of the process is related to the content and functions of various coagulation factors [27]. The results in this study suggested that the effect on coagulation may be mediated through different mechanisms from that of the antiplatelet effect.

Collagen and epinephrine play crucial role of platelet aggregation. When both are injected together, even at very low doses as used here, the synergism creates venous thrombin that results in fatal pulmonary embolism [29]. The induction of pulmonary embolism by collagen and epinephrine is considered to occur by a synergistic effect of these inducers. *R*-HPABA (medium and high dose) and *S*-HPABA (high dose) significantly increased the recovery rate compared with aspirin. In addition, the inhibitory effect of *R-*HPABA was higher than that of *S*-HPABA at medium and high dose. The inhibitory effect of *R-*/*S-*HPABA is consistent with its antiplatelet action. Platelet adhesion and aggregation are complicated processes. A variety of enzymes and receptors such as cyclooxygenase, thromboxane synthase, prostacyclin synthase, ADP receptor, fibrinogen receptor and thrombin receptor are involved in these processes. In the process of this experiment, collagen and epinephrine induced the platelet aggregation and then result in the pulmonary embolism. Therefore, the difference in collagen-epinephrine induced pulmonary thrombosis between *R*-HPABA and *S*-HPABA may resulted from the stereoselectivity of interactions between *R*-/*S*-HPABA and enzymes or receptors.

Injury of blood vessel endothelium can induce platelet adhesion and aggregation and meanwhile activate intrinsic and extrinsic coagulation systems, which leads to thrombosis [36]. The prostacyclin (PGI_2_) and thromboxane (TXA_2_) play important role in the process of antiplatelet aggregation. Because of the injury of vascular endothelial cell, synthesis of PGI_2_ is reduced and TXA_2_ in plasma is increased [37]. PGI_2_ and TXA_2_ have a short half-life and further convert to 6-keto-PGF1α and TXB_2_. However, TXA_2_ can reduce the synthesis of cyclic adenosine monophosphate (cAMP) and increase the concentration of Ca^2+^, which further leads to vasoconstriction, platelet aggregation and thrombosis [38].

TXA_2_ is produced from arachidonic acid through the COX-1 pathway in platelets. COX-1 play an important role in the production of TXA_2_ that amplifies platelet activation and induces vasoconstriction. In our study, *R-*/*S-*HPABA significantly inhibit the activity of COX-1. Furthermore, the effect of *S*-HPABA on the inhibition of COX-1 activity was higher than that of *R*-HPABA. A reason for this observation might be that the stereoselectivity of COX-1 was different between *R*-HPABA and *S*-HPABA. Additionally, *R-*/*S-*HPABA increased the plasma concentration of 6-keto-PGF1α and significantly decreased that of TXB_2_ (the metabolite of TXA_2_). These results indicated that the inhibition of platelet aggregation by *R-*/*S-*HPABA may be due to their inhibition of COX-1 enzyme leading to a reduced amount of TXA_2_ in platelets. In conclusion, the antithrombotic activity of *R-*/*S-*HPABA may be mediated by the inhibition of platelet aggregation at early stage of thrombosis formation.

## Conclusion

*R-*/*S-*HPABA have the same effects on antiplatelet aggregation and antithrombus compared with aspirin, however, it does not have severe gastrointestinal side effects like aspirin [20]. In addition, the antithrombotic activity of *R-*/*S-*HPABA may be mediated by the inhibition of platelet aggregation at early stage of thrombosis formation via inhibition of COX-1 activity including the inhibition of TXB_2_ formation. Moreover, the inhibitory effect of *R-*/*S-*HPABA on platelet aggregation was not due to cytotoxicity. Therefore, according to the *in vitro* and *in vivo* results, *R-*/*S-*HPABA are a promising antithrombus agent. Further experiments are required to study the accurate antiplatelet aggregation and antithrombus mechanisms.
